# Beneficial Effects of Fractions of *Nardostachys jatamansi* on Lipopolysaccharide-Induced Inflammatory Response

**DOI:** 10.1155/2014/837835

**Published:** 2014-03-30

**Authors:** Gi-Sang Bae, Kwang-Ho Heo, Sun Bok Choi, Il-Joo Jo, Dong-Goo Kim, Joon-Yeon Shin, Seung-Hee Seo, Kyoung-Chel Park, Dong-Sung Lee, Hyuncheol Oh, Youn-Chul Kim, Ho-Joon Song, Byung-Cheul Shin, Sung-Joo Park

**Affiliations:** ^1^Hanbang Body-Fluid Research Center, Wonkwang University, Iksan, Jeonbuk 540-749, Republic of Korea; ^2^Department of Rehabilitation Medicine of Korean Medicine, Spine & Joint Center, Pusan National University Korean Medicine Hospital, Yangsan 626-770, Republic of Korea; ^3^Department of Herbology, School of Oriental Medicine, Wonkwang University, Iksan, Jeonbuk 540-749, Republic of Korea; ^4^BK21 plus team, Professional Graduate School of Oriental Medicine, Wonkwang University, Iksan, Jeonbuk 540-749, Republic of Korea; ^5^Department of Beauty, Dongshin University, Naju, Jeonnam 252-17, Republic of Korea; ^6^College of Pharmacy, Wonkwang University, Iksan 570-749, Republic of Korea

## Abstract

It has been previously shown that *Nardostachys jatamansi* (NJ) exhibits anti-inflammatory properties against lipopolysaccharide (LPS) challenges. However, the potency of NJ constituents against LPS-induced inflammatory responses has not been examined. In this present study, we determined which NJ extract fractions exhibit inhibitory effects against LPS-induced inflammatory responses. Among the NJ fractions, NJ-1, NJ-3, NJ-4, and NJ-6 inhibited LPS-induced production of NO. The NJ-3, NJ-4, and NJ-6 fractions also inhibited the production of cytokines, such as IL-1**β**, IL-6, and TNF-**α**. However, NJ-1, NJ-3, NJ-4, and NJ-6 showed differential inhibitory mechanisms against LPS-induced inflammatory responses. NJ-1, NJ-3, and NJ-4 inhibited LPS-induced activation of c-jun NH_2_-terminal kinase (JNK) and p38 but did not affect activation of extracellular signal-regulated kinase (ERK) or NF-**κ**B. On the other hand, NJ-6 inhibited activation of MAPKs and NF-**κ**B. In addition, *in vivo* experiments revealed that administration of NJ-1, NJ-3, NJ-4, and NJ-6 reduced LPS-induced endotoxin shock, with NJ-6 especially showing a marked protective effect. Taken together, these results provide the evidence for the potential of selective NJ fractions against LPS-induced inflammation. Thus, it will be advantageous to further isolate and determine single effective compounds from these potent fractions.

## 1. Introduction

Inflammation is a complex immunologic response to harmful stimuli, including various pathogens, irritants, and infections [1]. Infectious agents, such as bacteria and proinflammatory cytokines, can activate macrophages, immune cells that are critically involved in the regulation of innate immunity, through certain receptors [2]. The interaction between Toll-like receptor (TLR)-4 and the ligand lipopolysaccharide (LPS) induces an intracellular signaling cascade that activates the mitogen-activated protein kinase (MAPK) family, extracellular signal-related kinase (ERK), p38, c-jun NH_2_-terminal kinase (JNK), and key proinflammatory transcription factors such as nuclear factor-kappa B (NF-*κ*B) [3]. Subsequent to TLR-4-LPS interaction, macrophages release various inflammatory mediators, such as nitric oxide (NO), and proinflammatory cytokines including interleukin (IL)-1*β*, IL-6, and tumor necrosis factor alpha (TNF-*α*) [4].

*Nardostachys jatamansi* (NJ) is widely used as a bitter tonic and antispasmodic [5]. It has been reported that the root of NJ contains various sesquiterpenes, including jatamansic acid, and jatamansone, lignans, and neolignans. We previously reported that aqueous extract of NJ is effective in protecting against inflammatory challenges [6–10], particularly against LPS-induced inflammation and endotoxin shock [7]. In particular, one fraction of NJ extract (fraction 4) demonstrated a protective effect against cerulein-induced acute pancreatitis [11]. However, although many of our studies have shown the anti-inflammatory activities of NJ, it is not yet known which particular compound of NJ has the potential to inhibit LPS-induced inflammation. Therefore, to take one step closer to actual bioactive compounds from NJ, we used NJ fractions, which have many bioactive compounds. In this study, we performed* in vitro* and* in vivo* analyses to examine whether the fractions of NJ have potential inhibitory effects against LPS-induced inflammation in murine peritoneal macrophages and in an animal model of LPS-induced endotoxin shock. Moreover, to elucidate a potential molecular anti-inflammatory mechanism, we examined the activation of MAPKs and NF-*κ*B.

## 2. Materials and Methods

### 2.1. Chemicals and Reagents

RPMI-1640, fetal bovine serum (FBS), penicillin, and streptomycin were obtained from Gibco BRL (Grand Island, NY, USA). Enzyme-linked immunosorbent assay (ELISA) antibodies for detection of mouse IL-1*β*, IL-6, and TNF-*α* were purchased from R&D Systems (Minneapolis, MN, USA). LPS from* Escherichia coli* 055:B5 was purchased from Sigma-Aldrich Chemical (St. Louis, MO, USA). Antibodies against total and phospho-specific MAPKs (ERK 1/2, JNK, and p38) were obtained from Cell Signaling Technology (Beverly, MA, USA). I*κ*-B*α* monoclonal antibody and peroxidase-conjugated secondary antibody were purchased from Santa Cruz Biotechnology, Inc. (Santa Cruz, CA, USA). Prestained sodium dodecyl sulfate-polyacrylamide gel electrophoresis markers were obtained from Bio-Rad (Hercules, CA, USA). Trizol reagent and polymerase chain reaction (PCR) kits were purchased from Invitrogen Corporation (Carlsbad, CA, USA).

### 2.2. Plant Materials

The roots of NJ were purchased from a standard commercial source (Omni Herb, Seoul, Korea). The herb's identity was confirmed at Wonkwang University. Voucher specimens (number 02-03-29) were deposited at the College of Oriental Medicine Herbarium of Wonkwang University. The NJ roots were prepared by decocting the dried prescription of herbs (100 g) with boiling distilled water (1 L) for approximately 2 h. The water extract was frozen at −80°C and freeze dried to be powdered (7.35 g, 7.35 w/w%).

### 2.3. Preparation of NJ Fractions

**Scheme 1:**
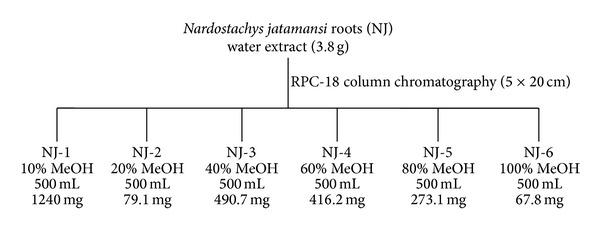
The scheme of NJ fractions.

The water extract (3.8 g) was subjected to octadecyl functionalized silica gel flash column (5 × 20 cm; 63–200 *μ*m particle size) chromatography (Scheme 1). Elution was carried out by stepwise gradient with 500 mL aliquots of MeOH in H_2_O (starting from 10% and followed by 20%, up to 100% at 20% increments), yielding 6 fractions (NJ1: 1.24 g; NJ2: 79.1 mg; NJ3: 490.7 mg; NJ4: 416.2 mg; NJ5: 273.1 mg; and NJ6: 67.8 mg).

### 2.4. HPLC Sample Preparation and HPLC Conditions

**Scheme 2:**
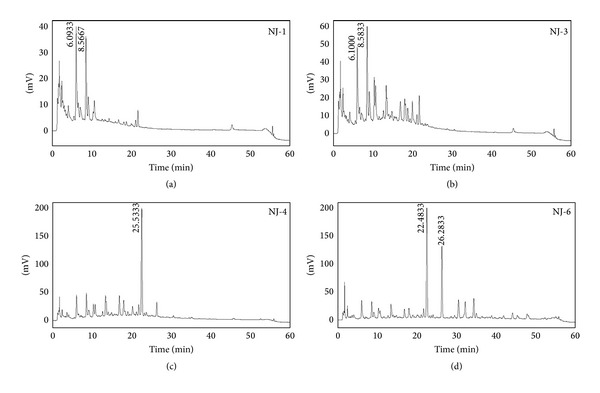
The HPLC of NJ biological fractions.

For HPLC analysis, the chromatographic system consisted of a pump (3000 HPLC pump; Dionex Association, USA), a UV detector (Photodiode array detector; Dionex Association), and an autosampler (Waters Association, USA). A hydrosphere C_18_ column (4.6 mm × 250 mm, 5 *μ*m) was used. The mobile phase was composed of (A) water and (B) acetonitrile, with an applied gradient of 10% B increasing to 60% B in 50 min and 60% B increasing to 100% B in 2 min, and then the system was equilibrated for 8 min with the 100% B conditions (total 60 min). Detection of the peaks was performed at 254 nm and sensitivity was set at 0.5 AUFs. The injection volume was 20 *μ*L, and the flow rate was 1.0 mL/min. A standard solution was prepared by dissolution in distilled methanol (10 *μ*g/10 mL). The solution of biological active fraction was filtered through a 0.45 *μ*m membrane filter and subjected to HPLC (Scheme 2).

### 2.5. Peritoneal Macrophage Culture

Thioglycollate (TG) elicited peritoneal macrophages were harvested 4 d after intraperitoneal injection of 3 mL TG. Peritoneal lavage was performed using 8 mL of RPMI supplemented with 10% heat-inactivated FBS. After incubation for 3 h, nonadherent cells were removed and the medium was changed for adherent cells. Cells were then treated with LPS in the absence or presence of NJ fractions.

### 2.6. MTT Assay

Cell viability was assayed using a modified colorimetric technique that is based on the ability of live cells to convert the tetrazolium compound 3-(4,5 dimethylthiazol)-2,5-diphenyl tetrazolium bromide (MTT) into purple formazan crystals. MTT (5 mg/mL) was dissolved in Krebs-Henseleit buffer (115 mM NaCl, 3.6 mM KCl, 1.3 mM KH_2_PO_4_, 25 mM NaHCO_3_, 1 M CaCl_2_, and 1 M MgCl_2_). Fifty microliters of the mixture was added to each well. After 30 min of incubation at 37°C, the suspension was removed and formazan crystals were dissolved in 200 *μ*L of dimethyl sulfoxide. Aliquots of cells were seeded in wells of a 96-well plate in duplicate and assayed at 540 nm using a micro-ELISA plate reader. The number of NJ fraction-treated viable cells was expressed as a percentage of the control (untreated cells) maintained for the same time period.

### 2.7. Measurement of NO Concentration

Murine peritoneal macrophages (2 × 10^5^ cells/well) were pretreated with NJ fractions for 1 h and then stimulated with LPS (500 ng/mL) for 24 h. To measure NO concentration, 100 *μ*L aliquots were removed from conditioned media and incubated with an equal volume of Griess reagent at room temperature for 10 min. The absorbance at 540 nm was then measured.

### 2.8. ELISA

Murine peritoneal macrophages (1 × 10^6^ cells/well) were stimulated with 500 ng/mL of LPS and/or various concentrations of NJ fraction for 24 h. Culture supernatants were collected and stored at −80°C until use. Cytokine levels in supernatants were determined using a commercial system (R&D Systems) according to the manufacturer's instructions. The ELISA was devised by coating 96-well plates with antibodies specific for IL-1*β*, IL-6, and TNF-*α*. Coated plates were washed with PBS containing 0.05% Tween-20. All reagents used in this assay were incubated overnight at 4°C. Recombinant IL-1*β*, IL-6, and TNF-*α* were diluted and used as a standard. Serial dilutions starting at 20 ng/mL were used to establish the standard curve. Assay plates were exposed sequentially to biotinylated mouse IL-1*β*, IL-6, and TNF-*α*, avidin peroxidase, and a substrate solution of 2,2′-azino-bis-(3-ethylbenzthiazoline-6-sulfonic acid) (ABTS) containing 30% hydrogen peroxide. The plates were read at 405 nm.

### 2.9. RNA Quantification

Total cellular RNA was isolated using Trizol (Invitrogen, Carlsbad, CA, USA) according to manufacturer's instructions. Total RNA (1 *μ*g) was converted to cDNA by 200 units of reverse transcriptase and 500 ng of oligo-dT primer in 50 mM Tris-HCl (pH 8.3), 75 mM KCl, 3 mM MgCl_2_, 10 mM DTT, and 1 mM dNTPs at 42°C for 1 h. The reaction was stopped by heating at 70°C for 15 min.

### 2.10. Real-Time RT-PCR

TaqMan quantitative RT-PCR was performed with a 7700 sequence detection system according to the manufacturer's instructions (Applied Biosystems, Foster City, CA, USA). For each sample, triplicate test reactions and a control reaction lacking reverse transcriptase were analyzed for expression of the gene of interest and results were normalized to those for the housekeeping gene hypoxanthine-guanine phosphoribosyltransferase (HPRT). Arbitrary expression units were calculated by dividing expression of the gene of interest by ribosomal protein HPRT mRNA expression. PCR was performed at an annealing temperature of 60°C with 40 amplification cycles. The commercial forward, reverse, and probe oligonucleotide primers for multiplex real-time TaqMan PCR were purchased from ABI (Applied Biosystems, Foster City, CA, USA).

### 2.11. Western Blot

Whole cell lysates were prepared by boiling peritoneal macrophages in a sample buffer (62.5 mM Tris-HCl (pH 6.8), 2% sodium dodecyl sulfate, 20% glycerol, and 10% 2-mercaptoethanol). Proteins in the cell lysates were then separated by 10% SDS-PAGE and transferred to a nitrocellulose membrane. The membrane was then blocked with 5% skim milk in PBS-Tween-20 for 2 h at room temperature and incubated overnight with antibodies against phosphorylated ERK 1/2, phosphorylated JNK, phosphorylated p38, and I*κ*-B*α*. After washing three times, each blot was incubated with a secondary antibody for 1 h, and immunoreactive bands were visualized using an enhanced chemiluminescence detection system (Amersham, Piscataway, NJ, USA) according to the manufacturer's recommended protocol.

### 2.12. Immunostaining

Peritoneal macrophages were plated in a chamber slide and incubated with 500 ng/mL of LPS for 1 h at 37°C. The cells were fixed in 4% paraformaldehyde for 30 min at RT and washed three times with PBS. The cells were treated with 0.1% Triton X-100 for 15 min at room temperature. After washing, the cells were incubated with a blocking serum for 1 h and incubated overnight with a 1 : 100 dilution of primary NF-*κ*B p65 antibody (Santa Cruz Biotechnology, Santa Cruz, CA, USA). The cells were then washed and incubated with a 1 : 2000 dilution of Alexafluor 568-conjugated goat anti-mouse IgG (Invitrogen, USA) for 2 h in a dark room. For nuclear staining, the cells were incubated with a 1 : 1000 dilution of DAPI for 5 min. The slide was finally washed and mounted for microscopic examination. Staining with NF-*κ*B p65 antibody exhibited red fluorescence, which was detected by fluorescence microscopy.

### 2.13. Animal Model of LPS-Induced Endotoxin Shock

Endotoxin shock was induced in 6- to 8-week-old female C57BL/6 mice by intraperitoneal (i.p.) injection of bacterial endotoxin (LPS from* E. coli* serotype O55:B5, 37.5 mg/kg). At 1 h after NJ fraction administration, LPS (37.5 mg·kg^−1^) was injected intraperitoneally. Survival was monitored for 120 h. Animal use and relevant experimental procedures were conducted in accordance with the National Institutes of Health (NIH) Guidelines for the Care and Use of Laboratory Animals. All experiments were approved by the Animal Care Committee of Wonkwang University.

### 2.14. Statistical Analysis

The results are expressed as the mean ± S.E. of independent experiments. Two-way ANOVA was used to analyze the statistical significance of results among groups. If results were found to be statistically significant,* post hoc* analysis was performed using the Duncan method as a multiple comparison among groups. All statistical analyses were performed using SPSS, version 10.0 statistical analysis software.

## 3. Results

### 3.1. Cytotoxicity of NJ Fractions in Peritoneal Macrophages

**Figure 1:**
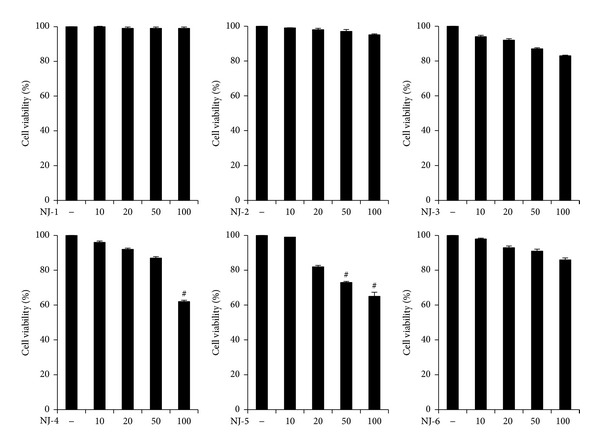
Cytotoxic effects of NJ fractions on murine peritoneal macrophages. The effects of NJ fractions on cell viability were measured by MTT assay. Peritoneal macrophages were incubated with or without NJ fractions at indicated doses (*μ*g/mL) for 24 h. The values are mean ± S.E. of three independent experiments. ^#^ *P* < 0.001 versus saline.

The first approach to study the biological activity of any compound or plant extract is to ensure lack of effect on cellular metabolism. To determine whether fraction of NJ affects cell viability, peritoneal macrophages were incubated for 24 h with varying concentrations of extract (*μ*g/mL), and cell viability was evaluated by MTT assay. NJ-1, NJ-2, NJ-3, and NJ-6 did not demonstrate any significant cytotoxic effects on peritoneal macrophages. However, at a relatively higher dose, NJ-4 and NJ-5 showed significant cytotoxicity (^#^ *P* < 0.001) (Figure 1). Therefore, because higher levels of NJ-4 (≥100 *μ*g/mL) and NJ-5 (≥50 *μ*g/mL) could affect cellular metabolism, we excluded data of NJ-4 and NJ-5 at these concentrations.

### 3.2. Effects of NJ Fractions on LPS-Induced NO Production

**Figure 2:**
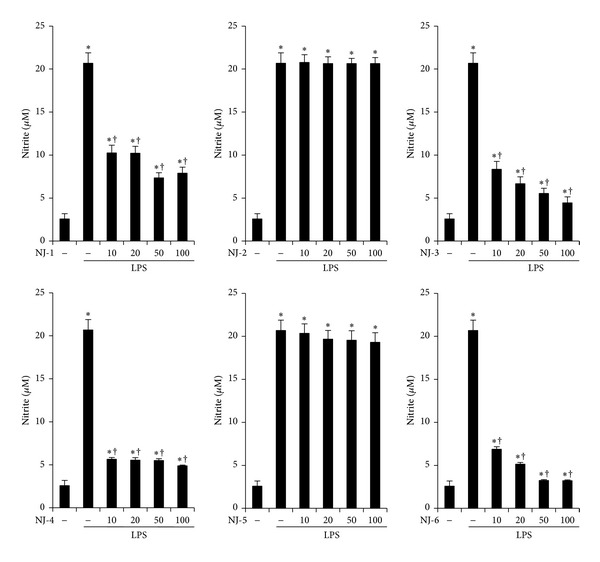
Effects of NJ fractions on LPS-induced NO production. Peritoneal macrophages were pretreated with NJ fractions at indicated doses (*μ*g/mL) for 1 h and then stimulated with LPS (500 ng/mL) for 24 h. The effects of NJ fractions on LPS-induced NO production were measured by Griess assay. The values are mean ± S.E. of three independent experiments. *P < 0.05 versus saline. ^+^ *P* < 0.05 versus LPS.

Next, to examine the anti-inflammatory effect of NJ fractions, we measured NO production. Murine peritoneal macrophages were pretreated with the indicated concentrations of NJ fractions for 1 h and then stimulated with LPS (500 ng/mL) for 24 h. As shown in Figure 2, LPS stimulation increased NO production. However, pretreatment with NJ-1, NJ-3, NJ-4, and NJ-6 significantly inhibited LPS-induced production of NO but not with NJ-2 and NJ-5 (Figure 2).

### 3.3. Effects of NJ Fractions on LPS-Induced Proinflammatory Cytokine Production

**Figure 3:**
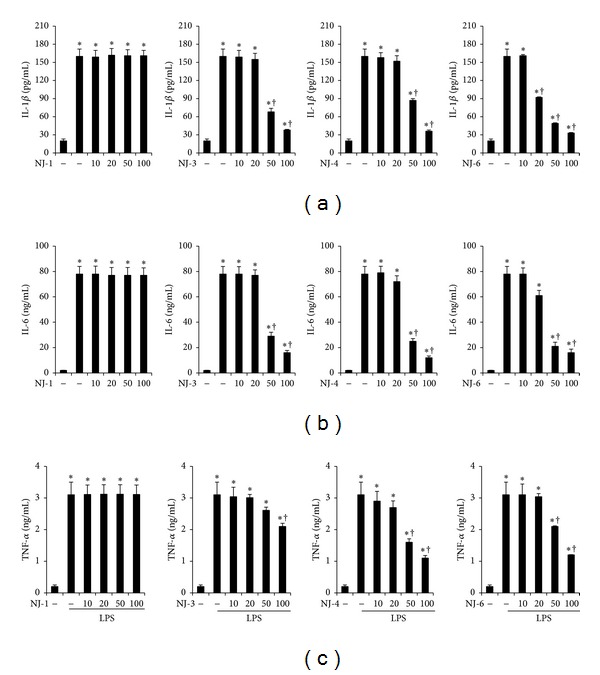
Effects of NJ fractions on LPS-induced cytokine productions. Peritoneal macrophages were treated with NJ fractions (*μ*g/mL) for 1 h and then stimulated with LPS at indicated time points. The supernatants were harvested and the levels of (a) IL-1*β*, (b) IL-6, and (c) TNF-*α* were measured by ELISA. The values are mean ± S.E. of three independent experiments. *P < 0.05 versus saline. ^+^ *P* < 0.05 versus LPS.

**Figure 4:**
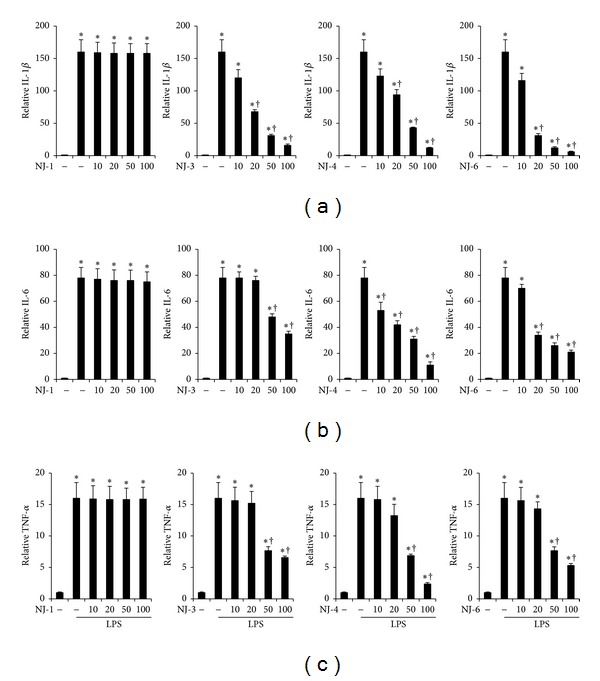
Effects of NJ fractions on mRNA levels of LPS-induced cytokine expression. Peritoneal macrophages were treated with NJ fractions (*μ*g/mL) for 1 h and then stimulated with LPS at indicated time points. Cells were collected for real-time RT-PCR. Total RNA (1 *μ*g) was prepared for the real-time RT-PCR of (a) IL-1*β*, (b) IL-6, and (c) TNF-*α*. The values are mean ± S.E. of three independent experiments. *P < 0.05 versus saline. ^+^ *P* < 0.05 versus LPS.

To examine the effects of NJ fractions on LPS-induced proinflammatory cytokine production, peritoneal macrophages were pretreated with NJ fractions at the indicated concentrations for 1 h and then stimulated with 500 ng/mL of LPS for 24 h. In particular, we treated cells with NJ-1, NJ-3, NJ-4, and NJ-6 fractions, which demonstrated inhibition of NO production. As shown in Figures 3 and 4, protein and mRNA levels of IL-1*β*, IL-6, and TNF-*α* were increased by LPS stimulation. However, pretreatment with NJ-3, NJ-4, and NJ-6 inhibited the production of IL-1*β*, IL-6, and TNF-*α* significantly but not with NJ-1 fraction (Figures 3 and 4).

### 3.4. Effects of NJ Fractions on the Activation of MAPKs and NF-*κ*B

**Figure 5:**
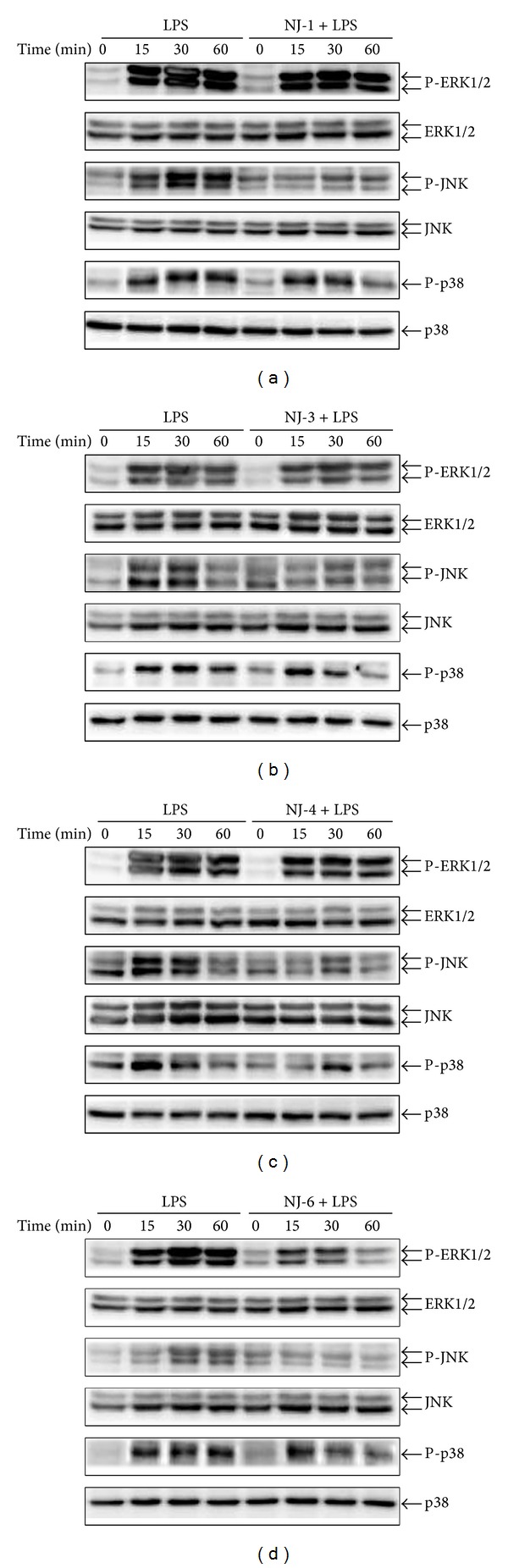
Effects of NJ fractions on MAPKs activation. ((a)–(d)) Peritoneal macrophages were treated with NJ fractions (100 *μ*g/mL) for 1 h and then stimulated with LPS (500 ng/mL) at indicated time points. Cells were harvested for western blot. Total cellular proteins (20 *μ*g) were resolved by SDS-PAGE, transferred to PVDF membrane, and detected with phospho-specific ERK 1/2 (42/44 kDa), JNK (46/54 kDa), and p38 (43 kDa) antibody. ERK, JNK, and p38 were used as a loading control. A representative western blot of three experiments is shown.

**Figure 6:**
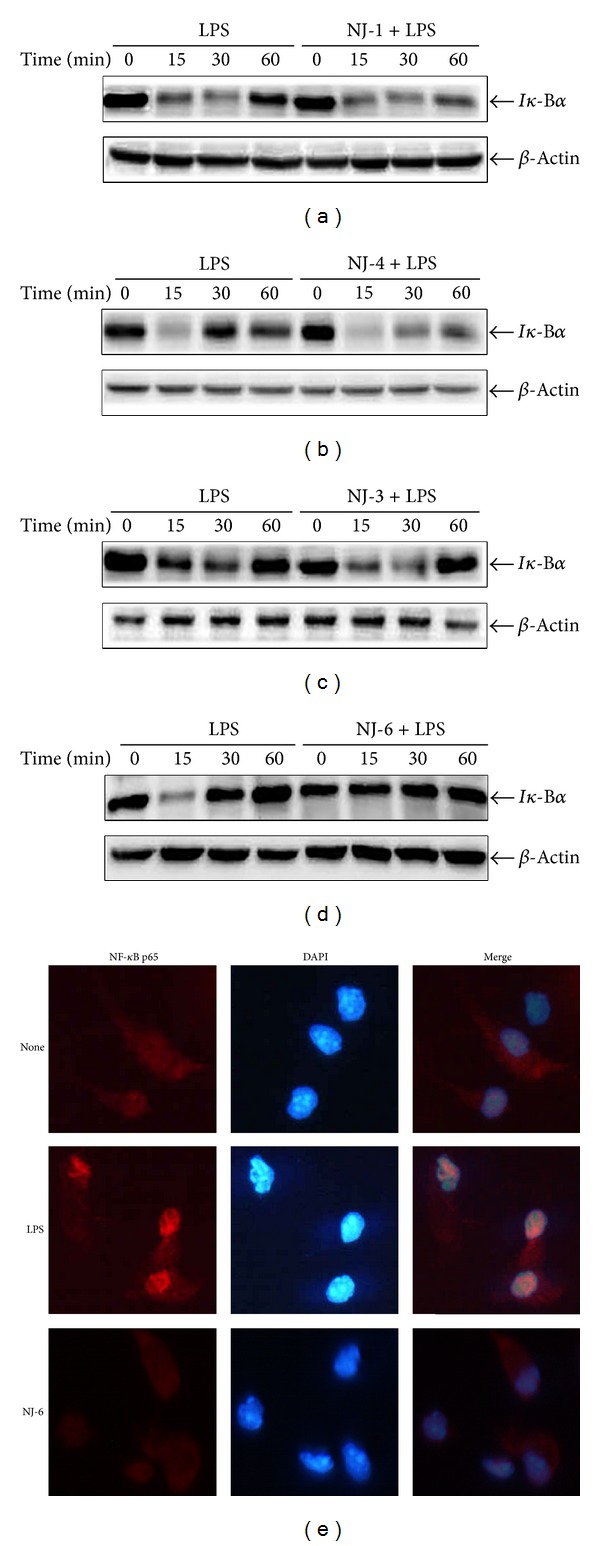
Effects of NJ fractions on NF-*κ*B activation. ((a)–(d)) Peritoneal macrophages were treated with NJ fractions (100 *μ*g/mL) for 1 h and then stimulated with LPS (500 ng/mL) at indicated time points. The cells were harvested for western blot. Total cellular proteins (20 *μ*g) were resolved by SDS-PAGE, transferred to PVDF membrane, and detected with I*κ*-B*α* (40 kDa) antibody. *β*-Actin was used as loading control. A representative western blot of three experiments is shown. (e) Peritoneal macrophages were treated with NJ fractions (100 *μ*g/mL) for 1 h and then stimulated with LPS (500 ng/mL) for 1 h. Then, nuclear translocation of NF-*κ*B p65 was examined using immunostaining. NF-*κ*B p65 was shown as red, and the nucleus was shown as blue (DAPI). The values are means ± S.E. of three independent experiments. *P < 0.05 versus saline. ^+^ *P* < 0.05 versus LPS.

To examine inhibitory mechanism(s) of NJ fractions on production of NO and cytokines, MAPK and NF-*κ*B were measured. The cells were pretreated with NJ-1 (100 *μ*g/mL), NJ-3 (100 *μ*g/mL), NJ-4 (50 *μ*g/mL), and NJ-6 (100 *μ*g/mL) for 1 h and then stimulated with 500 ng/mL of LPS for 0, 15, 30, or 60 min. Pretreatment with NJ-1, NJ-3, or NJ-4 significantly inhibited the LPS-induced activation of JNK and p38, but not ERK 1/2 (Figures 5(a)–5(c)). In addition, pretreatment with NJ-6 significantly inhibited the LPS-induced activation of ERK 1/2, JNK, and p38 (Figure 5(d)). To determine whether NJ fractions inhibit LPS-induced NF-*κ*B activation, we examined degradation of I*κ*-B*α* and NF-*κ*B p65 translocation. The NJ-1, NJ-3, and NJ-4 fractions did not inhibit the degradation of I*κ*-B*α* (Figures 6(a)–6(c)). However, NJ-6 inhibited the degradation of I*κ*-B*α* and translocation of NF-*κ*B p65 into nucleus, suggesting that NJ-6 may influence LPS-induced NF-*κ*B activation (Figures 6(d) and 6(e)).

### 3.5. Effects of NJ Fractions on LPS-Induced Endotoxin Shock

**Figure 7:**
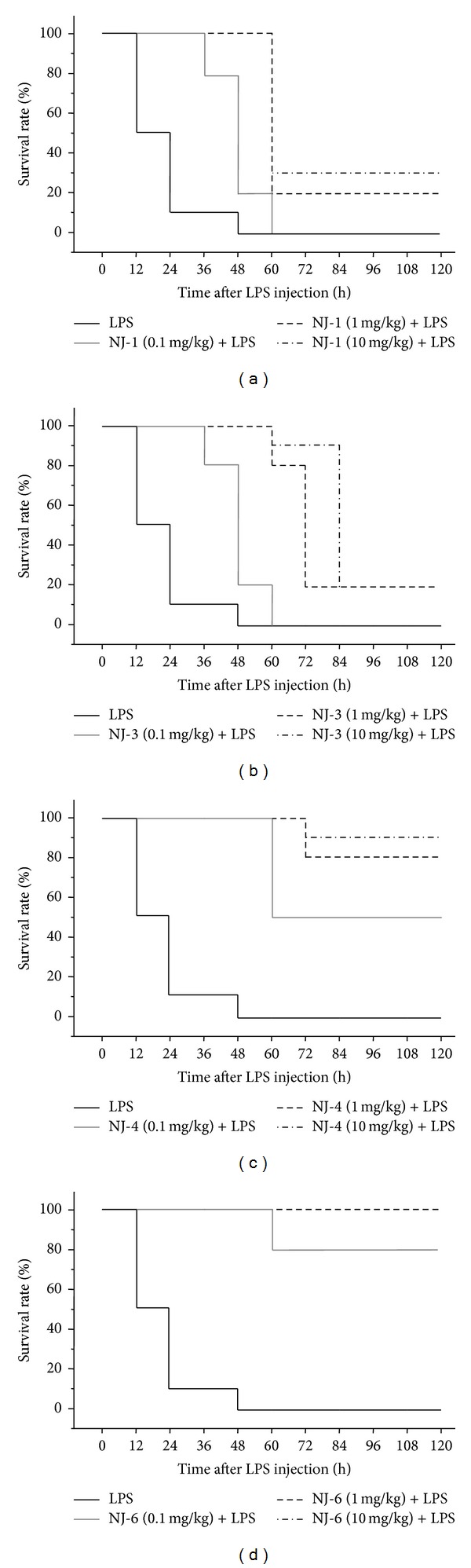
Effect of NJ fractions on LPS-induced endotoxin shock. ((a)–(d)) Endotoxin shock was induced in 6–8-week-old female C57BL/6 mice, as described in Section 2. NJ fractions were administered intraperitoneally to mice at 0.1, 1, or 10 mg/kg 1 h before LPS injection. Then, LPS (37.5 mg/kg) was injected to induce endotoxin shock. The survival rates of endotoxin shock mice were monitored for survival for 120 h. This figure shows data for 10 mice per group.

To verify effects of NJ fractions* in vivo*, we used a model LPS-induced endotoxin shock. NJ fractions (NJ-1, NJ-3, NJ-4, or NJ-6) were injected at the indicated doses. After 3 h, a lethal dose of LPS (37.5 mg/kg) was administered intraperitoneally. The survival rate was recorded every 12 h after LPS treatment. Generally, LPS-injected septic mice died within 48 h. Consistent with* in vitro* experiments, NJ fractions exhibited strong anti-inflammatory activities* in vivo*, as shown by significant inhibition of LPS-induced death in the treated mice (Figure 7). In particular, NJ-4 and NJ-6 showed the most dramatic protection against LPS-induced endotoxin shock; these fractions prevented nearly all harmful effects of LPS (Figures 7(c) and 7(d)).

## 4. Discussion

Recently, our laboratory reported the beneficial effects of NJ on various diseases, such as endotoxin shock, pancreatitis, and diabetes [6–10]. Particularly in endotoxin shock, NJ showed a marked preventive effect and strong therapeutic potential against LPS challenge [7]. Although many of our studies have indicated the benefits of NJ on inflammatory diseases, which compounds of NJ could exhibit anti-inflammatory properties are unknown. Thus, in this paper, bringing more bright insight to identify the constituent compounds from NJ, we selected and assorted effective fractions that would possess anti-inflammatory potential. Overall, we obtained six fractions from NJ. Using the fourth fraction, we evaluated the protective effects on cerulein-induced acute pancreatitis (AP). However, the sixth fraction remained to be examined for potential to prevent an inflammatory response to stimuli such as LPS. Therefore, in this study, we used six fractions isolated from NJ to examine the anti-inflammatory activity against LPS on murine macrophages.

Generally, sepsis is a life threatening condition caused by the body's response to bacterial products, such as endotoxin, which is often present with severe fever, shock, and respiratory damage [12]. Partially due to this phenomenon, sepsis is often regarded as an uncontrolled inflammatory response [12]. In the context of sepsis, TNF-*α*, IL-1*β*, IL-6, and IL-8 are the most frequently altered cytokines [13]. When injected into animals, these cytokines recapitulate many clinical and laboratory features of sepsis, supporting the concept that sepsis represents a “cytokine storm.” Thus, regulation of cytokines may prove valuable for the prevention and treatment of sepsis. In this study, just as the total extract of NJ inhibited the production of cytokines [7], fractions 3, 4, and 6 also inhibited the production of cytokines (Figures 3 and 4). These results suggest that the ability of NJ to inhibit cytokine production may originate from fractions 3, 4, and/or 6.

Using the fractions that were found to be effective against LPS, we examined activation of MAPKs and NF-*κ*B as possible regulatory mechanisms. Generally, it has been reported that the production of proinflammatory mediators is modulated by the MAPKs and NF-*κ*B pathways. Three major groups of MAPKs include the ERK, JNK, and p38, which are all activated by phosphorylation [14–16]. NF-*κ*B activation occurs mainly through the degradation of I*κ*B, thereby leading to subsequent release of NF-*κ*B dimers [17]. Subsequently, NF-*κ*B dimers translocate from the cytoplasm to the nucleus and activate the transcription of multiple *κ*B-dependent target genes [17]. In this study, we examine the phosphorylation of MAPKs and degradation of I*κ*-B*α*. NJ-1, NJ-3, and NJ-4 inhibited the phosphorylation and activation of JNK and p38 but not ERK1/2. However, NJ-6 inhibited not only the activation of ERK1/2, JNK, and p38 but also degradation of I*κ*-B*α*. These results suggest that inhibition of NO and cytokine production by NJ fractions is primarily through MAPKs or NF-*κ*B pathways.

In this study, we isolated two beneficial fractions of NJ. The first one was NJ-4, which could show similar effects to NJ total extract. NJ-4 inhibited cytokine production and LPS-induced endotoxin shock similar to NJ total extract [7]. Furthermore, the regulatory mechanisms were similar; NJ total extract inhibited only MAPKs, but NJ-4 inhibited the JNK and p38. Secondly, NJ-6 showed dramatic inhibition of LPS-induced inflammatory response. Indeed, NJ-6 inhibited the cytokine production and LPS-induced endotoxin shock dramatically. However, NJ-6 differed from total extract mechanistically; NJ-6 inhibited the degradation of I*κ*-B*α* and translocation of NF-*κ*B p65, which was not regulated by the NJ total extract. Further isolation of the NJ-4 and NJ-6 fractions could potentially yield a single compound demonstrating inhibitory activity resembling that of NJ total extract.

In conclusion, this study demonstrated which fractions of NJ exhibit inhibitory activity against LPS-induced inflammation. Among the 6 fractions isolated, NJ-1, NJ-3, NJ-4, and NJ-6 showed the inhibition of NO, and NJ-3, NJ-4, and NJ-6 showed the inhibition of cytokines. In addition, NJ-4 and NJ-6 exhibited dramatic prevention against LPS-endotoxin shock. These results suggest that NJ-4 and NJ-6 may be effective and beneficial candidates to ameliorate LPS-induced inflammation.

## Abbreviations

ELISA: Enzyme-linked immunosorbent assay
ERK: Extracellular signal-regulated kinase
FBS: Fetal bovine serum
IL: Interleukin
JNK: c-jun NH_2_-terminal kinase
LPS: Lipopolysaccharide
MAPKs: Mitogen-activated protein kinases
MTT: 3-(4,5 Dimethylthiazol)-2,5-diphenyl tetrazolium bromide
NF: Nuclear factor
NJ: * Nardostachys jatamansi*
NO: Nitric oxide
PCR: Polymerase chain reaction
TG: Thioglycollate
TLR: Toll-like receptor
TNF: Tumor necrosis factor.
